# Hypnotism

**Published:** 1888-12

**Authors:** 


HYPNOTISM is now attracting much attention' especially in France. It must also come into extensive use in this country. More than at any previous age of the world the power of mental impression, and the influence of the nervous system are beginning to be understood and appreciated by the profession. In this number of our Journal may be seen an interesting selection relative to the relief of a number of cases of dysmenorrhœa by what is called suggestion to the hypnotic. As far back as 1883 the managing editor of this journal taught in his lectures at the Southern Medical College the power of mental impression as shown in mesmerism, which is but another name for the phenomena now called hypnotism, and which has been recently so prominently brought before the profession by Charcot, of Paris.

For a long time it had been customary with the profession to ridicule mes erism as a humbug and a delusion, but now it is everywhere acknowledged to be a truth, and one which is fraught with great possibilities of good or evil. In a paper read before the Medical Association of Georgia in April 1887, with title of *Duality of the Brain—A Theory of Mind Reading and Slate Writing*. Dr. Word, the editor of the SOUTHERN MEDICAL RECORD, promulgated a theory upon this subject which presents many plausible points in connection with these mysterious, nervous phenomena. Since the publication of this paper many contributions have appeared on hypnotism from German and French experts, and a few in America The late Richard Proctor a short while before his death, wrote a paper advancing views on the double action of the brain almost identical with those expressed by Dr. Word in the paper referred to. From 80 to 90 per cent. of persons experimented upon appear to be susceptible to the influence of mesmerism or hypnotism and when brought under the influence may be relieved by suggestion, as it is called, of almost any nervous disease, as of neuralgia; nervous headache, and such functional disorders as may be largely due to nervous derangements. Suggestion simply means a confident statement to the hypnotized patient that he is well, or will be relieved at a given time. If the disease is periodical it will only be necessary to say to the patient that when the next period arrives for a return of the malady it will not appear as usual, but that he will be perfectly free from the attack.

The power of hypnotism should not be exercised for trivial purposes and the time will come when it will be necessary to impose legal restriction upon its excise. For it has been proven that suggestions for evil are as potent as suggestions for good, and hence such a power may be abused by unprincipled parties to the injury and even to the ruin of those who may come under their influence. Females are more subject to the mesmeric influence than males, and children under twelve or fourteen years most so. We doubt not that within a few years time hypnotism will attract great attention in the medical world, and under proper restraints and protection will be extensively practiced, and will constitute an agency of great and unprecedent power in the treatment of nervous affections.

